# Perceptions of Spectacle Use Among Undergraduate Students in Oman: Visual Symptoms, Convenience, and Disadvantages

**DOI:** 10.3390/healthcare13192525

**Published:** 2025-10-06

**Authors:** Janitha Plackal Ayyappan, Hilal Alrahbi, Gopi Vankudre, Zoelfigar Mohamed, Virgina Varghese, Sabitha Sadandan

**Affiliations:** College of Health Sciences, University of Buraimi, Al Buraimi P.O. Box 890, Oman; hilal.h@uob.edu.om (H.A.); gopi.v@uob.edu.om (G.V.); virgina.v@uob.edu.om (V.V.); sabitha.s@uob.edu.om (S.S.)

**Keywords:** perception of spectacle use, undergraduate students, visual symptoms, convenience, disadvantages

## Abstract

**Background:** Globally, uncorrected refractive errors are recognized as the primary cause of visual impairment and blindness. According to a report by the World Health Organization (WHO), providing spectacle lenses at an affordable cost remains a significant challenge, particularly for underprivileged populations in developing countries. This challenge contributes to the low compliance with spectacle wear worldwide. However, the benefits of wearing spectacles are influenced by the perceptions of the population regarding spectacle use. **Methods:** A quantitative, cross-sectional survey-based study was conducted at a superior educative center in Oman, the University of Buraimi. Participants were recruited from the four major colleges, namely, the College of Health Sciences (COHS), College of Business (COB), College of Engineering (COE), and College of Law (COL), and the Center for Foundation Studies (CFS). This study was conducted over the period from 18 December 2022 to 18 December 2023. Essential data were collected using an electronic questionnaire facilitated by the Google platform. The initial section of the questionnaire outlines this study’s objectives and its benefits to the community. The digital survey comprises three sections: the first section addresses the sociodemographic profile of the participants; the second section explores perceptions related to spectacles; and the third section examines visual symptoms associated with spectacle wear. In this study, a pre-tested survey was administered following consultation with a panel of three subject matter experts who reviewed the clarity and content validity of the test items. Data analyses were performed using descriptive statistics, and linear regression was applied to assess the effect of socioeconomic profile on perceptions of spectacles. Additionally, data entry, processing, and analysis were conducted using SPSS 25 software. The overall mean score for spectacle-related visual symptoms was 2.51 ± 0.75, indicating a moderate level of symptom occurrence. **Results:** A total of 415 participants (N = 415) were included in this study, comprising 133 males (32.0%) and 282 females (68.0%). The most prominent symptoms related to spectacle perception were “light sensitivity” and “eye pain”, with mean values of 3.03 ± 1.30 and 3.04 ± 1.25, respectively. Additionally, 249 participants (60%) reported moderate concern regarding spectacle-related visual symptoms. Among female participants, 118 (41.8%) exhibited little concern about visual symptoms associated with spectacle wear, whereas this was observed in 25.6% of male participants. Descriptive statistics indicated the mean perceived spectacle-related disadvantages score measured on a scale of 0 to 4 was 2.88 ± 1.16 (57.69% ± 23.15% in percentages), reflecting a moderate perception of such disadvantages. The linear regression model demonstrated statistical significance, as indicated by the likelihood ratio chi-square = 199.194 (df = 15, *p* < 0.001). The most significant predictor was study major (χ^2^ = 72.922, *p* < 0.001). **Conclusions:** The present study indicates that undergraduate students generally exhibit a low perception of the disadvantages associated with wearing spectacles. Randomized sampling should be preferred in future studies to the convenience sampling technique. The most frequently reported visual symptoms include “light sensitivity and eye pain” among spectacle wearers. Therefore, it is imperative to implement health education programs and foundational studies across colleges to address these issues among undergraduate university students.

## 1. Introduction

Uncorrected refractive errors are globally recognized as the leading cause of reversible visual impairment and blindness if they are corrected at an appropriate time [1]. An emmetropic eye requires no additional optical power to focus parallel incoming rays of light on the macula while accommodation is relaxed. Frequently, however, this is not the case: refractive errors (myopia, hyperopia, or the different types of astigmatism, classified according to specific characteristics) may occur [2]. When their magnitude significantly affects the quality of the retinal image, uncorrected visual acuity diminishes (for distance or near, depending on the nature of the ametropia and the age of the patient), and the individual requires spectacles to perform daily activities normally. Uncorrected refractive errors are globally recognized as the leading cause of visual impairment and blindness. Although reversible with optical correction, they can significantly affect quality of life. These errors affect approximately 2.3 billion individuals worldwide, with 153 million experiencing vision impairment as a result [3,4,5]. The prevalence of refractive errors varies widely across regions and populations. In Asia, particularly China, myopia has reached alarming levels, with nearly 57% of children and adolescents affected and rates surpassing 90% in high school students [6]. In Europe, a recent meta-analysis estimated an overall prevalence of 23.5%, ranging from 11.9% in Finland to almost 50% in Sweden, with clear increases across age group [7]. In North America, NHANES data indicate that 44.7% of adults over 20 years are myopic, while global estimates suggest similar rates to Europe, around 25–30% [8]. In Africa, although prevalence remains lower (4.7% in children and adolescents), it has doubled in the last decade and is projected to rise substantially by 2050 [9,10]. In Latin America, the MIOPUR study in Colombia reported hyperopia in 32.3% and myopia in 12.9%, with opposing age-related trends [11]. In Australia, longitudinal cohort studies, including the Raine Study, confirm a progressive rise in myopia during young adulthood and highlight risk factors such as reduced sunlight exposure, female sex, and parental history [12,13]. In response to this public health challenge, the World Health Organization (WHO) has urged member states to ensure the provision of affordable spectacle lenses to those in need. However, this remains a significant challenge in developing countries, where poor compliance with spectacle wear is prevalent [14,15,16]. Recent studies from the Middle East have reported the prevalence of refractive errors among youth, with myopia, hyperopia, and astigmatism affecting 30%, 21%, and 24% of the population, respectively [17,18]. The prevalence of refractive errors among undergraduate medical students in Saudi Arabia, reported at 58.7%, is an alarming indicator of the increasing incidence of uncorrected refractive errors, particularly myopia, necessitating urgent public health intervention [19]. Similarly, research conducted in developing countries indicates that the affordability of spectacles is significantly low among underprivileged groups due to poor socioeconomic conditions and low literacy levels [20]. Spectacle correction is recognized as one of the most cost-effective and primary methods for correcting refractive errors. However, if uncorrected refractive errors persist, they can negatively impact academic performance, particularly among the youth population, such as undergraduate university students, ultimately affecting their quality of life [1,21,22]. Consistent use of spectacle lenses enhances vision, thereby increasing productivity and positively impacting an individual’s overall quality of life [10]. However, there exists a social stigma associated with the use of spectacle lenses among younger adults [23,24,25,26,27,28]. The primary reasons for not wearing spectacles include a lack of awareness regarding refractive errors and the benefits of spectacles in alleviating visual symptoms, as well as cosmetic concerns and myths suggesting that wearing spectacles may lead to blindness or visual impairment [24,28]. Additionally, there are misconceptions that wearing glasses may reduce the likelihood of finding a life partner and that spectacle lenses are intended solely for older individuals [24,26,29]. This could be attributed to the social stigma.

Furthermore, spectacles play a vital role in enhancing vision and are an essential component of eye care services, particularly for the youth population. However, the perception of spectacle-related visual symptoms has not been extensively studied in the literature, especially within the Sultanate of Oman. Therefore, this study aims to investigate the perceptions of spectacle lens use among undergraduate students in Oman, focusing on their associated visual symptoms, convenience, and disadvantages in this context.

## 2. Materials and Methods

### 2.1. Study Design

A cross-sectional study utilizing a questionnaire was conducted at the University of Buraimi to evaluate undergraduate students’ perceptions of spectacle lens wear, along with the associated visual symptoms, conveniences, and disadvantages, within the Sultanate of Oman.

### 2.2. Study Setting

This study was conducted at the University of Buraimi, which comprises four colleges, namely, Health Sciences, Business, Engineering, and Law, and a Center for Foundation Studies. This research received approval from the Research and Ethics Committee of the College of Health Sciences, University of Buraimi, Oman, with Ethics Approval No. AY 22-23 COHS-R07, and adhered to the principles of the Declaration of Helsinki. This study was conducted from 18 December 2022 to 18 December 2023. Pre-structured online survey-based test items were employed to collect the necessary data. The primary rationale for selecting the four major colleges and the Center for Foundation Studies for data collection was the diverse enrollment of participants in various programs from different regions of Oman, including both rural and urban areas.

### 2.3. Participants

Participation in the study was contingent upon the participants’ willingness to accept the survey, which was regarded as consent to participate. However, receiving the electronic survey was entirely optional for participants. The initial section of the survey provided an overview of the research objectives and outlined the benefits to the community. The inclusion criteria for this study encompassed all students registered in the four colleges and the Center for Foundation Studies, aged between 17 and 40 years. Conversely, students who declined to receive the electronic survey and those over 40 years of age were excluded from this study.

### 2.4. Data Sources/Study Instrument Used

The data required for this study were collected through a pre-structured survey, which was developed by the researchers following an extensive review of the literature [15,16] and consultations with experts to ensure this study’s objectives were achieved and to minimize errors during data collection. The clarity and content validity of the test items were verified by a panel of three experts in the field. The survey comprises three sections: demographic profile, socioeconomic status, and test items related to perceptions of spectacles and associated visual symptoms. Participants rated their perceptions of spectacle-related visual symptoms on a scale from 0 to 4, where 0 signifies “strongly disagree” and 4 signifies “strongly agree”. Similarly, perceptions regarding the convenience and disadvantages of spectacle use were also rated on the same scale. An electronic version of the questionnaire was distributed anonymously to all willing participants. The test items were designed to elicit precise responses, with logical sequencing applied to ensure that subsequent responses followed in a coherent manner. The final version of the survey was administered using Google Forms.

### 2.5. Study Size

Participants were recruited using convenience sampling techniques. Voluntary acceptance to participate in this research was considered as an indication of a willingness to participate.

### 2.6. Statistical Methods

This exploratory study conducted an analysis of data pertaining to participants’ academic discipline, gender, age, geographical location, occupational profile, and parental literacy status. Descriptive statistics were utilized to evaluate perceptions concerning spectacles and visual symptoms. A linear regression analysis was employed to investigate the impact of socioeconomic background on perceptions of spectacles. Data entry, processing, and analysis were executed using SPSS 25 software.

### 2.7. Ethical Considerations

Ethical Approval: This study was granted approval by the Research and Ethics Committee of the College of Health Sciences, University of Buraimi, Oman, under Ethics Approval No. AY 22-23 COHS-R07 and adhered to the principles outlined in the Declaration of Helsinki. Participants were presented with an electronic survey, and their voluntary participation and completion of the survey were regarded as informed consent to engage in this research.

## 3. Results

### 3.1. Demographic Profile of the Participants

This study included a total of 415 participants, with 133 (32.0%) males and 282 (68.0%) females.

### 3.2. Descriptive Statistics for the Perception Towards Spectacle Wear-Related Visual Symptoms Among the Participants

This study evaluated the visual symptoms associated with spectacle wear, using a 5-point Likert scale. The results showed that light sensitivity and eye pain were the most common reported symptoms, with mean scores of 3.03 ± 1.30 and 3.04 ± 1.25, respectively. Other symptoms had lower mean scores, indicating varying levels of agreement among participants. The overall mean score for spectacle-related visual symptoms was 2.51 ± 0.75, suggesting a moderate level of symptom occurrence. Additionally, the percentage score for these perceived symptoms was 15.18% ± 14.97%, highlighting the variability in participants’ experiences. These findings show the importance of addressing the visual discomfort in spectacle wearers to enhance their overall visual experience. More details in Table 1.

### 3.3. Level of Concern Towards Spectacle Wear-Related Visual Symptoms Among the Participants

Level of concern towards spectacle wear-related visual symptoms among the participants was statistically significant (*p* = 0.01). The majority, 249 (60%), of participants expressed moderate concern about spectacle-related visual symptoms. Moreover, 118 (41.8%) females were more likely to report little concern compared to males (25.6%), while 94 (70.7%) males were more likely to report moderate concern compared to females (55.0%). Severe concern was relatively low among both genders, with only 14 (3.4%) of the total sample representing this level of concern, as shown in Table 2.

### 3.4. Perception Towards Spectacle Usage-Related Convenience Among the Participants

Table 3 showed the overall mean score for spectacle usage-related convenience was 2.72 ± 0.97 (54.33% ± 19.31%, in percentages), indicating a moderate level of convenience.

### 3.5. Perceived Spectacle Usage-Related Convenience Observed Among the Participants

Overall, the majority, 239 (57.6%), of participants, perceived spectacles as moderately convenient. In total, 18 (6.4%) females were more likely to report spectacle as highly convenient compared to males (0.8%), while 97 (72.9%) males were more likely to report moderate convenience compared to females (50.4%). Of the total population, 157 (37.8%) reported less convenience with spectacles, with 122 females (43.3%) more likely to report this level compared to males (26.3%), for more details see Table 4.

### 3.6. Perception Spectacle Usage-Related Disadvantages Among the Participants

In Table 5, the overall mean score for the spectacle-related disadvantages was 2.88 ± 1.16 (57.69% ± 23.15%, in percentages), indicating a moderate level of perceived disadvantages, as in Table 3.

### 3.7. Perceived Spectacle Usage-Related Disadvantages Among the Participants

A substantial proportion of participants (185, 44.6%) perceived spectacles as highly disadvantageous. A higher percentage, 90 (67.7%), of males reported spectacles as highly disadvantageous compared to females (33.7%). Of the total sample, 51 (12.3%) reported a moderate level of disadvantages, with 41 (14.5%) females more likely to report this level compared to males (7.5%), as shown in Table 6.

### 3.8. Impact of Sociodemographic Factors on Perceived Convenience and Disadvantages Related to Spectacle Usage

A generalized linear regression analysis suggested that educational background, gender, age and parental socio-educational status significantly influence students’ perceived convenience related to spectacles. The overall model was statistically significant as indicated by a likelihood ratio chi-square = 199.194 (df = 15, *p* < 0.001). The most significant predictor was study major (χ^2^ = 72.922, *p* < 0.001), male participants scored significantly lower than females (χ^2^ = 9.904, *p* = 0.002), participants in the age group of 31 to 40 had notably lower scores (χ^2^ = 78.708, *p* < 0.001), urban populations scored slightly higher (χ^2^ = 3.991, *p* = 0.046), participants with working mothers scored higher (χ^2^ = 4.193, *p* = 0.041), participants whose father had a lower literacy level scored higher (χ^2^ = 12.733, *p* = 0.002), and participants whose mothers had intermediate literacy status had higher scores (χ^2^ = 16.002, *p* < 0.001).

However, it is essential to note that, though statistically significant (*p* = 0.046), considering this borderline statistically significant value, the urban or rural location of the participants might suggest limited practical impact on students’ perceived convenience related to spectacles

In Table 7, the generalized linear regression analysis also indicated that younger age, study major, parental occupation, and literacy level significantly influence perceived spectacle usage-related disadvantages. The study major of business reported the greatest disadvantage (χ^2^ = 91.553, *p* < 0.001), younger age groups experienced substantially higher disadvantages (χ^2^ = 139.354, *p* < 0.001), and participants whose father had lower literacy reported greater disadvantages (χ^2^ = 6.995, *p* = 0.030).

## 4. Discussion

The present study aimed to investigate the perceptions of spectacle lens wear among undergraduate students and the associated visual symptoms, conveniences, and disadvantages in the Sultanate of Oman. Moreover, the findings discussed in this study are limited to the data collected from the University of Buraimi students, native to different regions of Oman. This study found that most participants were female students, comprising 282 (68.0%) of the total, while male participants accounted for 133 (32.0%) out of 415 total participants. This gender distribution may reflect the overall enrollment trend at the university, which shows a higher female population across all colleges.

The overall mean score for visual symptoms was 50.18% ± 14.97%. Notably, the perception of visual symptoms was predominantly reported as “eye pain” and “light sensitivity” while wearing spectacle lenses, with mean scores of 3.03 ± 1.30 and 3.04 ± 1.25, respectively. These findings suggest a need to address visual discomfort associated with spectacle wear. Other visual symptoms, such as headache, glare, blurry vision, ghost images, and difficulty with night vision, were observed to have lower mean scores, with varying levels of agreement among participants. These observations are supported by a study conducted by Adeoti et al., which reported that participants expressed perceived experiences of wearing spectacles, including headache and eye pain, and noted relief upon removing the glasses [30]. Similarly, a study reported that wearing glasses can alleviate symptoms such as headaches, eye strain, watering, and blurred vision, although this statement contrasts with our study’s observations, as the former primarily focused on perceptions related to binocular vision abnormalities, specifically eye muscle imbalances [31].

Further research into investigating the underlying cases of visual symptoms associated with spectacle wear could be useful.

Additionally, most study participants expressed a “moderate concern” (249, 60%) regarding visual symptoms while wearing spectacles. Interestingly, females exhibited “very little concern” (118, 41.8%) compared to males when wearing glasses. Similarly, the present study indicates that the convenience of spectacles is perceived at moderate levels of satisfaction, described as “easy to use, low maintenance, and affordable”, with an average score of 2.72 (54.33%). Our study also found that the female population considers spectacles “highly convenient” (18, 6.4%) compared to the male population, which is less than 1 (0.8%). Likewise, the degree of spectacle-related disadvantages varies from “highly disadvantageous, moderately disadvantageous, to slightly disadvantageous”, with a total mean score of 2.88 ± 1.16 (57.69% ± 23.15%), indicating a moderate level of perception regarding the disadvantages of spectacles. Among these, 90 (67.7%) of the male population expressed the concern of wearing spectacles as “highly disadvantageous” compared to females (33.7%). These findings are supported by a study conducted by Khandekar et al., which reported that complaints were more prevalent among males compared to females regarding spectacle use. However, this observation contrasts with the notion that females typically exhibit poor perception towards spectacle wear due to cosmetic concerns and discomfort caused by the temple length, which often irritates the ear area due to the headscarf coverage in the female population. Nonetheless, these findings require further investigation to substantiate [32].

The present study examines the influence of sociodemographic factors on perceptions regarding the disadvantages of spectacle use through a linear regression analysis. The variables considered in this analysis include participants’ education, gender, age, and the literacy status of their parents. The findings indicate that participants’ education and the sociodemographic status of their parents significantly affect spectacle use and its convenience.

The most significant predictor for perceived convenience related to spectacles was study major (χ^2^ = 72.922, *p* < 0.001), males scored lower than females (χ^2^ = 9.904, *p* = 0.002), participants aged between 31 and 40 had notably lower scores (χ^2^ = 78.708, *p* < 0.001), urban populations scored higher (χ^2^ = 3.991, *p* = 0.046), and participants with working mothers (χ^2^ = 4.193, *p* = 0.041), fathers having a lower literacy level (χ^2^ = 12.733, *p* = 0.002), mothers having intermediate literacy status (χ^2^ = 16.002, *p* < 0.001) had higher scores.

Notably, individuals majoring in business reported a “high disadvantage” in perceived spectacle use (χ^2^ = 91.553, *p* < 0.001), surpassing other majors. Similarly, younger age groups perceived greater disadvantages associated with spectacle wear (χ^2^ = 139.354, *p* < 0.001). Additionally, participants with lower paternal literacy also reported “high disadvantages” (χ^2^ = 6.995, *p* = 0.030) in their perceptions of spectacle wear. These findings are corroborated by a study conducted by Pavithra MB et al., which demonstrated that fathers with a poor educational background exhibited less compliance compared to those with higher literacy. The same study also revealed that mothers with lower literacy levels had fewer complaints than those with higher literacy regarding perceptions of spectacle wear. Furthermore, it was indicated in discussion that children with fathers possessing higher literacy levels reported more complaints than those with fathers possessing lower literacy levels [33].

### Study Limitations and Future Research

The present study acknowledges certain limitations. Firstly, the study population was recruited exclusively from the university demographic. Secondly, the age range of participants was restricted to 17–40 years. Thirdly, the study instrument was pretested and utilized based on the existing literature; however, sensitivity analysis is recommended in future studies. Most of the study participants were female, which may introduce gender bias. Lastly, the data collection was conducted solely through questionnaires. However, this study is strengthened by its large sample size (N = 415) and its focus on measuring perceptions of spectacle wear and visual symptoms, which are rarely assessed in Oman. Moreover, the lack of refractive error specification is an important methodological limitation, as perceptions could differ between myopia, hyperopia, and astigmatism. While it would indeed have been more complex to classify refractive error, a practical approach could have been to request that participants include the spectacle data from their optical prescription (i.e., spherical and cylinder for each eye), which can be easily classified using simple formulas (2). An additional limitation is that the type of refractive error was not recorded. Different ametropias may influence perceptions about spectacle use, as previously noted in the literature (Hutchinson et al. 2023 [34]; O’Leary et al. 2003 [35]; Sprunger et al. 2023 [36]). Although more complex, classification would have been feasible by asking participants to provide the spectacle data from their optical prescription (i.e., sphere and cylinder for each eye) and applying simple formulas (2).

Future research should prioritize conducting longitudinal studies across various universities in Oman to explore perceptions of spectacle use, related visual symptoms, and their associated advantages and disadvantages.

## 5. Conclusions

The present study identified low perception regarding the disadvantages associated with spectacle use. “Eye pain and light sensitivity” emerged as the most frequently reported visual symptoms under the perception of spectacle wear. Consequently, it is imperative to address this issue within university-level student populations to dispel myths and misconceptions related to spectacles and enhance comfort for spectacle wearers. Furthermore, health education initiatives should be conducted among targeted university groups to improve perceptions related to spectacle use. Randomized sampling should be preferred in future studies to the convenience sampling technique

## Figures and Tables

**Table 1:** Perception towards spectacle wear-related visual symptoms among the participants.

Descriptive Statistics				
	Minimum	Maximum	Mean (Out of 5)	Std. Deviation
Can wearing glasses (spectacles) cause any of the problems listed below? (Rate the factors listed below using a Likert scale with 4 being strongly agree and 0 being strongly disagree) [Light sensitivity]	0	4	3.03	1.30
Can wearing glasses (spectacles) cause any of the problems listed below? (Rate the factors listed below using a Likert scale with 4 being strongly agree and 0 being strongly disagree) [Headache]	0	4	2.34	1.00
Can wearing glasses (spectacles) cause any of the problems listed below? (Rate the factors listed below using a Likert scale with 4 being strongly agree and 0 being strongly disagree) [Glare]	0	4	2.19	0.97
Can wearing glasses (spectacles) cause any of the problems listed below? (Rate the factors listed below using a Likert scale with 4 being strongly agree and 0 being strongly disagree) [Eye pain]	0	4	3.04	1.25
Can wearing glasses (spectacles) cause any of the problems listed below? (Rate the factors listed below using a Likert scale with 4 being strongly agree and 0 being strongly disagree) [Blurry vision]	0	4	2.05	0.97
Can wearing glasses (spectacles) cause any of the problems listed below? (Rate the factors listed below using a Likert scale with 4 being strongly agree and 0 being strongly disagree) [Ghost images]	0	4	2.30	1.07
Can wearing glasses (spectacles) cause any of the problems listed below? (Rate the factors listed below using a Likert scale with 4 being strongly agree and 0 being strongly disagree) [Difficulty with night vision]	0	4	2.89	1.29
Can wearing glasses (spectacles) cause any of the problems listed below? (Rate the factors listed below using a Likert scale with 4 being strongly agree and 0 being strongly disagree) [Fluctuation of vision]	0	4	2.22	0.95
Mean score for spectacle-related visual symptoms	0.000	4.000	2.51	0.75
Percentage mean score for spectacle-related visual symptoms	0.00%	80.00%	50.18%	14.97%

**Table 2:** Level of concern towards spectacle wear-related visual symptoms among the participants.

	Gender							
Level of Perceived Concern for Spectacle-Related Visual Symptoms	Male		Female		Total		Pearson Chi-Square	
	N	%	N	%	N	%	Value	*p* Value
Little concern	34	25.6%	118	41.8%	152	36.6%	10.345	0.006
Moderate concern	94	70.7%	155	55.0%	249	60.0%		
Severe Concern	5	3.8%	9	3.2%	14	3.4%		
Total	133	100.0%	282	100.0%	415	100.0%		

**Table 3:** Perception towards spectacle usage-related convenience among the participants.

	Mean	Std. Deviation
Rate spectacle usage-related convenience for the following categories on a scale of 0 to 4, with 4 being the highest convenience and 0 being the least convenience [Easy to use]	2.48	1.08
Rate spectacle usage-related convenience for the following categories on a scale of 0 to 4, with 4 being the highest convenience and 0 being the least convenience [Least maintenance]	2.66	0.90
Rate spectacle usage-related convenience for the following categories on a scale of 0 to 4, with 4 being the highest convenience and 0 being the least convenience [Affordability]	3.02	1.58
Mean score of spectacle usage-related convenience	2.72	0.97
Percentage mean score of spectacle usage-related convenience	54.33%	19.31%

**Table 4:** Degree of perceived spectacle usage-related convenience observed among the participants.

			Gender				Total				
	Male		Male_95% CI	Female		Female_95% CI			Total_95% CI	Pearson Chi-Square	
Perceived level of spectacle usage-related convenience	N	%		N	%		N	%		Value	*p*-Value
Highly convenient	1	0.8%	(0.0%, 2.3%)	18	6.4%	(3.5%, 9.2%)	19	4.6%	(2.7%, 6.7%)	21.12	<0.001
Less convenience	35	26.3%	(18.8%, 33.8%)	122	43.3%	(37.6%, 48.9%)	157	37.8%	(33.3%, 42.4%)		
Moderate convenience	97	72.9%	(65.4%, 80.5%)	142	50.4%	(44.7%, 56.0%)	239	57.6%	(52.8%, 62.4%)		
Total	133	100.0%	—	282	100.0%	—	415	100.0%	—		

**Table 5:** Perception spectacle usage-related disadvantages among the participants.

	Minimum	Maximum	Mean	Std. Deviation
Rate the spectacle usage-related disadvantages for the following categories on a scale of 0 to 4, with 4 being strongly agree and 0 being strongly disagree [Maintenance issue]	0	4	2.78	1.35
Rate the spectacle usage-related disadvantages for the following categories on a scale of 0 to 4, with 4 being strongly agree and 0 being strongly disagree [Restriction on daily life]	0	4	2.88	1.26
Rate the spectacle usage-related disadvantages for the following categories on a scale of 0 to 4, with 4 being strongly agree and 0 being strongly disagree [Cosmetic concerns]	0	4	2.99	1.24
Mean spectacle usage-related disadvantages	0.00	4.00	2.88	1.16
Percentage spectacle usage-related disadvantages	0.00%	80.00%	57.69%	23.15%

**Table 6:** Degree of perceived spectacle usage-related disadvantages among the participants.

			Gender								
	Male		Male_CI	Female		Female_CI	Total		Total_CI	Pearson Chi-Square	
Level of spectacle usage-related disadvantages	N	%		N	%		N	%		Value	*p*-Value
Highly disadvantageous	90	67.7%	(59.4%, 75.2%)	95	33.7%	(28.4%, 39.4%)	185	44.6%	(39.8%, 49.4%)	42.265	<0.001
Hardly disadvantageous	33	24.8%	(18.0%, 32.3%)	146	51.8%	(46.1%, 57.4%)	179	43.1%	(38.3%, 48.0%)		
Moderately disadvantageous	10	7.5%	(3.0%, 12.0%)	41	14.5%	(10.6%, 18.8%)	51	12.3%	(9.2%, 15.4%)		
Total	133	100.0%		282	100.0%		415	100.0%			

**Table 7:** Impact of sociodemographic factors on perceived convenience and disadvantages related to spectacle usage.

Attribute	Spectacle-Related Disadvantages			Spectacle-Related Convenience		
	Wald Chi-Square	df	Sig.	Wald Chi-Square	df	Sig.
(Intercept)	837.058	1	<0.001	690.811	1	<0.001
Study Major	91.553	4	<0.001	72.922	4	<0.001
Gender	0.752	1	0.386	9.904	1	0.002
Age in years	139.354	3	<0.001	78.708	3	<0.001
Geographical location	2.756	1	0.097	3.991	1	0.046
Occupation of father	9.219	1	0.002	3.615	1	0.057
Occupation of mother	11.896	1	0.001	4.193	1	0.041
Father’s literacy status	6.995	2	0.030	12.733	2	0.002
Mother’s literacy status	1.300	2	0.522	16.002	2	<0.001

Dependent Variable: Percentage spectacle usage-related disadvantages. Model: (Intercept), 1. Study Major, 2. Gender, 3. Age in years, 4. Geographical location, 5. Occupation of father, 6. Occupation of mother, 7. Father’s literacy status, 8. Mother’s literacy status.

## Data Availability

The raw data underpinning the conclusions will be made available upon request to the corresponding author.

## Abbreviations

The following are the abbreviations used in this study:

WHO	World Health Organization
COHS	College of Health Sciences
COB	College of Business
COE	College of Engineering
COL	College of Law
CFS	Center for Foundation Studies
